# Epidemiological Survey of Tick-Borne Zoonotic Pathogens in Questing Ticks from the Qinghai-Tibetan Plateau of China

**DOI:** 10.3390/ani16182927

**Published:** 2026-09-17

**Authors:** Yihong Ma, Xiuping Li, Yingna Jian, Guanghua Wang, Yong Hu, Xiangyin Cai, Peilin Ye, Hushou Liu, Dengfeng Zhao, Xinming Duan, Yue Zhang, Masakatsu Taira, Thillaiampalam Sivakumar, Ken Maeda, Xuenan Xuan, Naoaki Yokoyama, Liqing Ma

**Affiliations:** 1 Qinghai Provincial Key Laboratory of Pathogen Diagnosis for Animal Diseases and Green Technical Research for Prevention and Control, Academy of Animal Sciences and Veterinary, Qinghai University, Xining 810016, China; 2 National Research Center for Protozoan Diseases, Obihiro University of Agriculture and Veterinary Medicine, Inada-cho, Obihiro 080-8555, Japan; 3 Animal Husbandry and Veterinary Station of Huzhu County, Haidong 810399, China; 4 Qinghai Haidong Center for Animal Disease Prevention and Control, Haidong 810600, China; 5 Animal Husbandry and Veterinary Station of Datong County, Xining 810800, China; 6 Animal Husbandry and Veterinary Station of Menyuan County, Haibei Tibetan Autonomous Prefecture 810399, China; 7 Nongfayuan (Hainan) Agricultural Development Co., Ltd., Sanya 572024, China; 8 Department of Veterinary Science, National Institute of Infectious Diseases, Japan Institute for Health Security, Shinjuku-ku, Tokyo 162-8640, Japan; 9 Research Center for Asian Infectious Diseases, Institute of Medical Science, University of Tokyo, Shirokanedai, Tokyo 108-8639, Japan

**Keywords:** Anaplasmataceae, *Babesia*, Qinghai, questing ticks, *Rickettsia*

## Abstract

Tick-borne zoonotic pathogens, including *Rickettsia*, *Anaplasma*, *Ehrlichia*, and *Babesia* spp., pose increasing risks to human and animal health. This study investigated tick species composition and associated zoonotic pathogens across high-(1500–3000 m) and very high-altitude (>3000–5500 m) zones of the Qinghai-Tibetan Plateau area (QTPA). A total of 1025 questing ticks were collected from 11 counties and identified as *Haemaphysalis qinghaiensis* (*n* = 316), *Dermacentor nuttalli* (*n* = 685), *D. silvarum* (*n* = 20), and *D. niveus* (*n* = 4). Tick composition showed clear zonal variation. In the high-altitude zone, all four species were present, with *H. qinghaiensis* as the dominant species. In contrast, only *Dermacentor* spp. were detected in the very high-altitude zone, where *D. nuttalli* predominated. PCR and sequence analyses detected DNA of multiple pathogens in 137/1025 (13.4%) ticks. *Rickettsia* was most prevalent (11.4%), followed by Anaplasmataceae (2.2%) and *Babesia* (0.2%). In the high-altitude zone, *H. qinghaiensis* harbored *R. raoultii*, *R. slovaca*, *Anaplasma phagocytophilum*, *A. bovis*, uncultured *Rickettsia*, *Anaplasma*, and *Ehrlichia* spp., and a *Babesia motasi*-like organism. In contrast, in the very high-altitude zone, *D. nuttalli* mainly carried *R. raoultii*, *R. slovaca*, *A. phagocytophilum*, *A. ovis*, and uncultured *Rickettsia* and *Ehrlichia* spp. In conclusion, tick species composition and associated pathogens varied between the altitude zones in QTPA. In particular, the predominance of human-biting *D. nuttalli* in the very high-altitude zone, together with detection of zoonotic agents, highlights the public health relevance of tick-borne infections in this region. Additional studies are essential to establish vector competence or transmission.

## Simple Summary

Tick-borne zoonotic pathogens can infect both animals and humans, but little is known about their distribution in high-altitude regions. In this study, we collected questing ticks from high-altitude (1500–3000 m) and very high-altitude (>3000–5500 m) zones of the Qinghai–Tibetan Plateau Area (QTPA), China, and screened them for zoonotic species of *Rickettsia*, *Anaplasma*, *Ehrlichia*, and *Babesia*. Tick species differed markedly between the two altitude zones. *Haemaphysalis qinghaiensis* was the predominant species in the high-altitude zone, whereas *Dermacentor nuttalli* predominated in the very high-altitude zone. Several zoonotic pathogens, including *Rickettsia raoultii*, *R. slovaca*, *Anaplasma phagocytophilum*, and *A. bovis*, were detected in the collected ticks. Zoonotic pathogens were detected in ticks from both altitude zones, suggesting potential human exposure to tick-borne pathogens throughout the study area, particularly in the very high-altitude zone where the human-biting tick *D. nuttalli* is predominant. However, detection of pathogen DNA does not establish vector competence or transmission. These findings provide a baseline for understanding the altitudinal distribution of questing tick species and associated zoonotic pathogens in the QTPA.

## 1. Introduction

Tick-borne zoonotic pathogens are an important and growing threat to global public health. Among them, species of *Rickettsia*, *Anaplasma*, *Ehrlichia*, and *Babesia*, which are transmitted by tick species belonging primarily to the genera *Ixodes*, *Haemaphysalis*, *Dermacentor*, *Amblyomma*, *Rhipicephalus*, and *Hyalomma*, are responsible for a wide range of human diseases [1,2,3,4]. Infections with these pathogens present with diverse clinical manifestations, ranging from mild febrile illness to severe, life-threatening conditions. *Rickettsial* infections in humans are mainly caused by members of the spotted fever group (SFG) and typhus group [3]. Similarly, within the family Anaplasmataceae, *Anaplasma phagocytophilum* causes human granulocytic anaplasmosis, whereas *Ehrlichia chaffeensis* and *E. ewingii* cause human ehrlichioses [4,5]. In addition, several *Babesia* species, including *B. microti*, *B. duncani*, *B. divergens*, *B. venatorum*, *B. odocoilei*, *B. crassa*-like, and *Babesia* sp. KO1 cause human babesiosis, which can be severe, particularly in elderly and immunocompromised individuals [6]. The clinical impact and rising incidence of human infections caused by these pathogens highlight the importance of effective control measures based on the epidemiological distribution [3,4,7].

As most pathogens are transmitted by specific tick species, the geographic distribution of tick-borne zoonotic diseases is closely linked to their tick vectors. Consequently, surveillance of questing ticks is a widely used approach to identify circulating pathogens and assess potential public health risks [8]. In China, diverse climatic conditions and geographic heterogeneity support a rich tick fauna, facilitating the circulation of multiple zoonotic pathogens [9]. Previous studies have reported the presence of several zoonotic tick-borne pathogens in China, including spotted fever group (SFG) *Rickettsia* spp. (e.g., *R. raoultii*, *R. sibirica*, *R. heilongjiangensis*, and *R. slovaca*), *Anaplasma phagocytophilum*, *Ehrlichia chaffeensis*, and zoonotic or potentially zoonotic *Babesia* species, such as *B. microti*, *B. divergens*, *B. venatorum*, and *B. crassa*-like pathogens [9,10]. Human infections caused by several of these pathogens have also been documented in China [6,9,11]. Rickettsial infections have been reported primarily in central and eastern regions of the country [12]. Human infections with *A. phagocytophilum*, *A. bovis*, and *Candidatus Ehrlichia erythraense* have also been reported in central and southeastern China [12,13]. Furthermore, more than 300 human babesiosis cases have been reported from at least 16 provinces in China, with *B. microti* being the predominant causative agent, whereas *B. venatorum* and *B. crassa*-like pathogens have been reported to cause human babesiosis in northeastern China [14]. However, most of these studies in China have focused on low-altitude regions, while high-altitude areas remain poorly investigated.

The Qinghai-Tibetan plateau area (QTPA) of China is a high-altitude, climate-sensitive region, where environmental conditions may influence tick distribution and pathogen transmission [15]. The QTPA harbors high-altitude-adapted tick species, such as *Dermacentor nuttalli* and *Haemaphysalis qinghaiensis* [16], whose distribution differs from that of tick species reported in low-altitude regions of China, potentially influencing pathogen transmission [17]. Previous studies in this region have identified several species of *Rickettsia* [7], *Anaplasma* [18], *Ehrlichia* [19], and *Babesia* [20] in ticks and animal hosts. However, these studies have mainly relied on ticks collected from infested animals [18,19], in which PCR positivity might result from ingested infected blood rather than true infection, limiting the ability to identify competent tick vectors.

Based on previously described altitudinal classification criteria and the established concept of the Death Zone, the QTPA can be broadly divided into four zones: high-altitude zone (1500–3000 m), very high-altitude zone (>3000–5500 m), extreme altitude (>5500–8000 m), and the death zone (>8000 m) [21,22]. The present study focused on the high-altitude and very high-altitude zones, where environmental conditions differ substantially. Compared with the high-altitude zone, the very high-altitude zone is characterized by lower temperatures, reduced oxygen availability, and a shorter growing season, which may influence tick survival, species composition, and pathogen transmission. Livestock production is an important agricultural activity in the QTPA, with yak, cattle, sheep, goats, and horses being among the major livestock species raised in the region [23]. Despite this, comparative studies examining tick species composition and associated pathogens across these altitudinal zones remain limited. Therefore, the present study aimed to determine the composition of questing tick species and the presence of zoonotic pathogens, including species of *Rickettsia*, *Anaplasma*, *Ehrlichia*, and *Babesia*, in the high- and very high-altitude zones of QTPA.

## 2. Materials and Methods

### 2.1. Collection, Morphological Identification, and Total DNA Extraction of Questing Ticks

**Figure 1:**
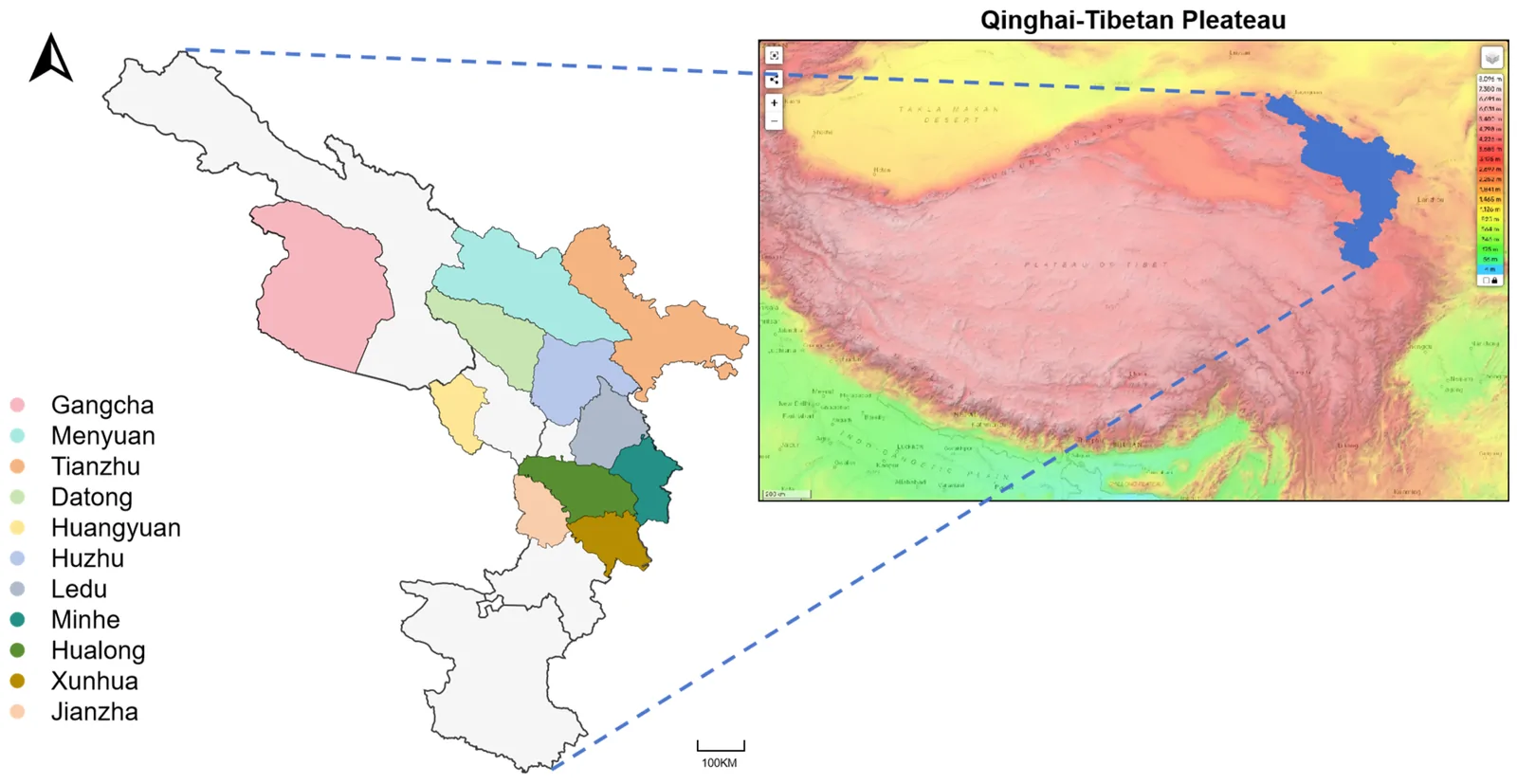
Map of the Qinghai-Tibetan Plateau area (QTPA) showing sampling locations of questing ticks. The base map was generated using DataV (https://datav.aliyun.com; accessed on 15 July 2026) and Topographic-Map (https://topographic-map.com; accessed on 15 July 2026). Colored symbols indicate the regions where tick samples were collected.

Questing ticks were collected from 11 different counties in the QTPA of China during March–May 2024 and 2025 (Figure 1) from the high-altitude zone and the very high-altitude zone. The sampling sites were inhabited by wildlife, such as wild yak, kiang, Tibetan antelope, plateau pika, and Himalayan marmot; livestock, such as yaks, Tibetan sheep, cattle, and horses; and humans, particularly local farmers and herders. Questing ticks were collected using the flagging method [24], in which a cloth was dragged over vegetation and examined periodically for attached ticks, from various habitat types, including alpine meadows and grasslands, tall grass and reed vegetation, shrub- and bush-dominated areas, and wet meadows and wetlands. After collection, ticks were preserved in 70% ethanol, and species were identified using a stereomicroscope, based on previously described morphological keys [25]. Genomic DNA samples were extracted from individual ticks using an ISOHAIR Kit (Nippon Gene, Toyama, Japan), following the manufacturer’s instructions, and then stored at −30 °C until further analysis.

### 2.2. PCR Detection of Rickettsia gltA, Anaplasmataceae groEL, and Babesia 18S rRNA Sequences

All DNA samples were screened with previously described nested PCR assays targeting the *gltA* gene of *Rickettsia* and the *groEL* gene of Anaplasmataceae, which are conserved among the respective pathogen groups, making them suitable targets for broad detection [26,27]. PCR primer sequences and amplicon sizes are provided in Table 1. Purified DNA samples from cultured *R. japonica* YH strain [28] and *A. phagocytophilum* HZ strain [29] were used as the positive controls in *Rickettsia*- and Anaplasmataceae- PCR assays, respectively. A reaction mixture without template DNA served as a negative control to monitor potential contamination. To minimize the risk of contamination, DNA extraction, preparation of the PCR mixture, addition of template DNA, and post-PCR analysis were performed in physically separated designated areas using dedicated pipettes. Aerosol-resistant filtered pipette tips were used throughout.

The DNA samples were also screened for zoonotic *Babesia* species using previously described conventional PCR assays, targeting the 18S rRNA, which is conserved among *Babesia* species but allows species identification upon sequencing [30]. Briefly, three 18S rRNA-based PCR assays were employed to detect three different groups of zoonotic *Babesia* species: (i) *B. microti* and *B. duncani* (BMD group); (ii) *B. divergens*, *B. venatorum*, and *B. odocoilei* (BDVO group); and (iii) *B. crassa*-like species and *Babesia* sp. KO1 (BCK group), as previously described [30]. *Babesia microti* DNA extracted from the blood of a mouse infected with the Munich strain [31], *B. divergens* DNA from an in vitro culture [32], and a synthetic 18S rRNA sequence representing *B. crassa*-like species (accession number: KU204785) were used as positive controls in BMD, BDVO, and BCK PCRs, respectively. A reaction mixture without template DNA served as a negative control. Positive samples were subjected to an additional PCR assay targeting a 361-bp fragment of the internal transcribed spacer 2 (ITS2) region of zoonotic *Babesia* parasites, using primers designed in the present study (Table 1). PCR reaction mixture and cycling conditions are similar to those for the *Babesia* PCR assays, except for the annealing temperature and extension time, which were adjusted to 52 °C and 60 s, respectively.

### 2.3. Sequencing and Phylogenetic Analyses

All PCR amplicons were excised from agarose gels and then subjected to direct Sanger sequencing at GENEWIZ (GENEWIZ Suzhou Co., Ltd., Suzhou, China). The resulting sequences were analyzed using the Basic Local Alignment Search Tool (BLAST, https://blast.ncbi.nlm.nih.gov/Blast.cgi?PROGRAM=blastn&PAGE_TYPE=BlastSearch&LINK_LOC=blasthome; accessed on 10 June 2026) in order to confirm their origins and determine the identity scores they share with reference sequences in GenBank. Newly generated sequences, together with those retrieved from GenBank, were aligned and analyzed using Molecular Evolutionary Genetics Analysis software (MEGA version X, [33]) to predict best-fitting substitution models. Finally, four maximum likelihood phylogenetic trees were constructed, based on the following substitution models: Tamura 3-parameter (*gltA* sequences of *Rickettsia*) [34], Hasegawa-Kishino-Yano (*groEL* sequences of Anaplasmataceae) [35], Jukes-Cantor (18S rRNA sequences of *Babesia*) [36], and Kimura 2-parameter (ITS2 sequences of *Babesia*) [37].

### 2.4. Statistical Analyses

Differences in tick species composition between the two altitude zones were evaluated using Pearson’s chi-square test of independence. Because two tick species had small expected frequencies, a Monte Carlo simulation test was additionally performed to obtain a more reliable significance assessment. The Monte Carlo test was based on 1,000,000 random simulations of contingency tables under the null hypothesis while maintaining the observed marginal totals. Statistical analyses were performed using R software (v 4.5.1, R Foundation for Statistical Computing, Vienna, Austria). A two-sided *p* value < 0.05 was considered statistically significant.

## 3. Results

### 3.1. Composition of Questing Ticks Collected in the QTPA of China

A total of 1025 questing ticks were collected from 11 different counties in the QTPA of China, including 364 ticks from the high-altitude zone and 661 ticks from the very high-altitude zone. Morphological examination identified four tick species: *Haemaphysalis qinghaiensis* (*n* = 316), *Dermacentor nuttalli* (*n* = 685), *Dermacentor silvarum* (*n* = 20), and *Dermacentor niveus* (*n* = 4) (Figure S1, Table 2). Of the collected *H. qinghaiensis*, 113 were males, 143 were females, and 60 were nymphs. *D. nuttalli* comprised 231 males and 454 females, with no nymphs collected. *D. silvarum* comprised 7 males and 13 females, whereas *D. niveus* comprised 2 males and 2 females; no nymphs were collected for either species. No larvae were collected during the survey.

In the high-altitude zone, all four tick species were detected, with *H. qinghaiensis* being predominant (316/364, 86.8%), followed by *D. nuttalli* (37/364, 10.2%), *D. silvarum* (9/364, 2.5%), and *D. niveus* (2/364, 0.5%). In contrast, *H. qinghaiensis* was absent in the very high-altitude zone, where only three *Dermacentor* spp. were detected. Among these, *D. nuttalli* was overwhelmingly predominant (648/661, 98.0%), followed by *D. silvarum* (11/661, 1.7%) and *D. niveus* (2/661, 0.3%) (Table 2). Tick species composition differed significantly between the high- and very high-altitude zones (Pearson’s χ^2^ = 846.18, df = 3, *p* < 0.001). Because some expected cell frequencies were small, a Monte Carlo test was additionally performed and likewise demonstrated a significant association between tick species composition and altitude zone (*p* < 0.001).

### 3.2. Positivity of Rickettsia gltA, Anaplasmataceae groEL, and Babesia 18S rRNA in Collected Ticks

PCR screening detected *Rickettsia*, Anaplasmataceae, and *Babesia* DNA in questing ticks collected from the high-altitude zone, whereas only *Rickettsia* and Anaplasmataceae DNA were detected in the very high-altitude zone. In the high-altitude zone, 71/364 (19.5%) ticks were positive for *Rickettsia gltA*, 18/364 (4.9%) for Anaplasmataceae *groEL*, and 2/364 (0.5%) for *Babesia* 18S rRNA (Table 2). DNA of *Rickettsia* spp. was detected in all four tick species, including *H. qinghaiensis* (60/316, 19.0%), *D. nuttalli* (7/37, 18.9%), *D. silvarum* (3/9, 33.3%), and *D. niveus* (1/2, 50.0%). In contrast, Anaplasmataceae DNA was detected only in *H. qinghaiensis* (17/316, 5.4%) and *D. silvarum* (1/9, 11.1%), whereas *Babesia* DNA was detected exclusively in *H. qinghaiensis* (2/316, 0.6%).

In the very high-altitude zone, 46/661 (7.0%) ticks were positive for *Rickettsia* and 5/661 (0.8%) for Anaplasmataceae, whereas no *Babesia* DNA was detected (Table 2). All positive samples were obtained from *D. nuttalli*, with detection rates of 7.1% (46/648) for *Rickettsia* and 0.8% (5/648) for Anaplasmataceae. Overall, the pathogen detection rates and pathogen group diversity were higher in the high-altitude zone than those of the very high-altitude zone. Notably, *H. qinghaiensis*, which was detected only in the high-altitude zone, was the only tick species positive for all three pathogen groups.

### 3.3. Rickettsia Species Detected in Collected Ticks

**Figure 2:**
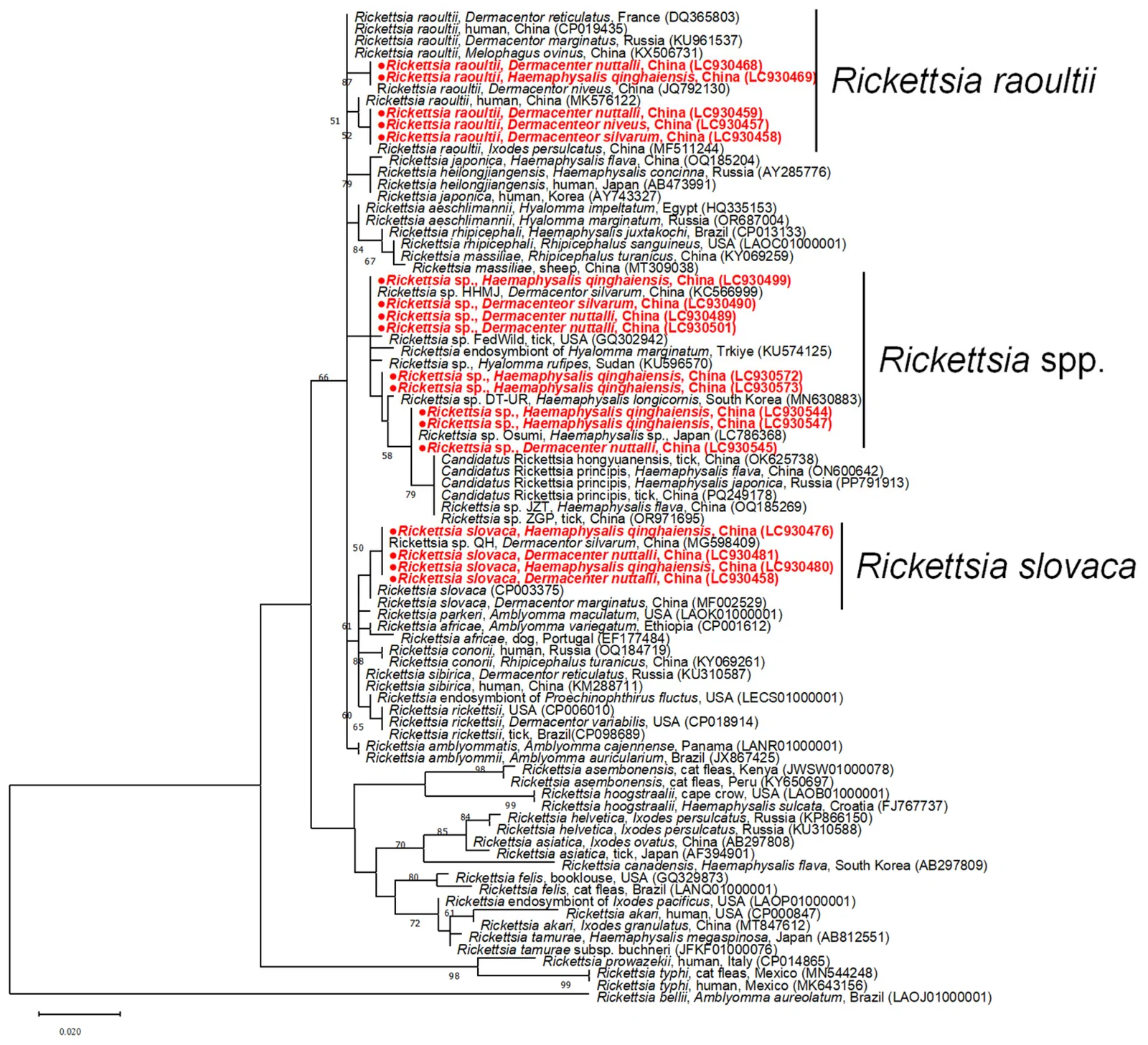
Phylogenetic analysis of *Rickettsia* based on *gltA* gene sequences. A maximum likelihood tree was constructed using *gltA* sequences obtained in the present study, together with reference sequences retrieved from GenBank. Sequences generated in the present study, shown in red, clustered with *Rickettsia slovaca*, *Rickettsia raoultii*, and uncultured *Rickettsia* spp.

**Figure 3:**
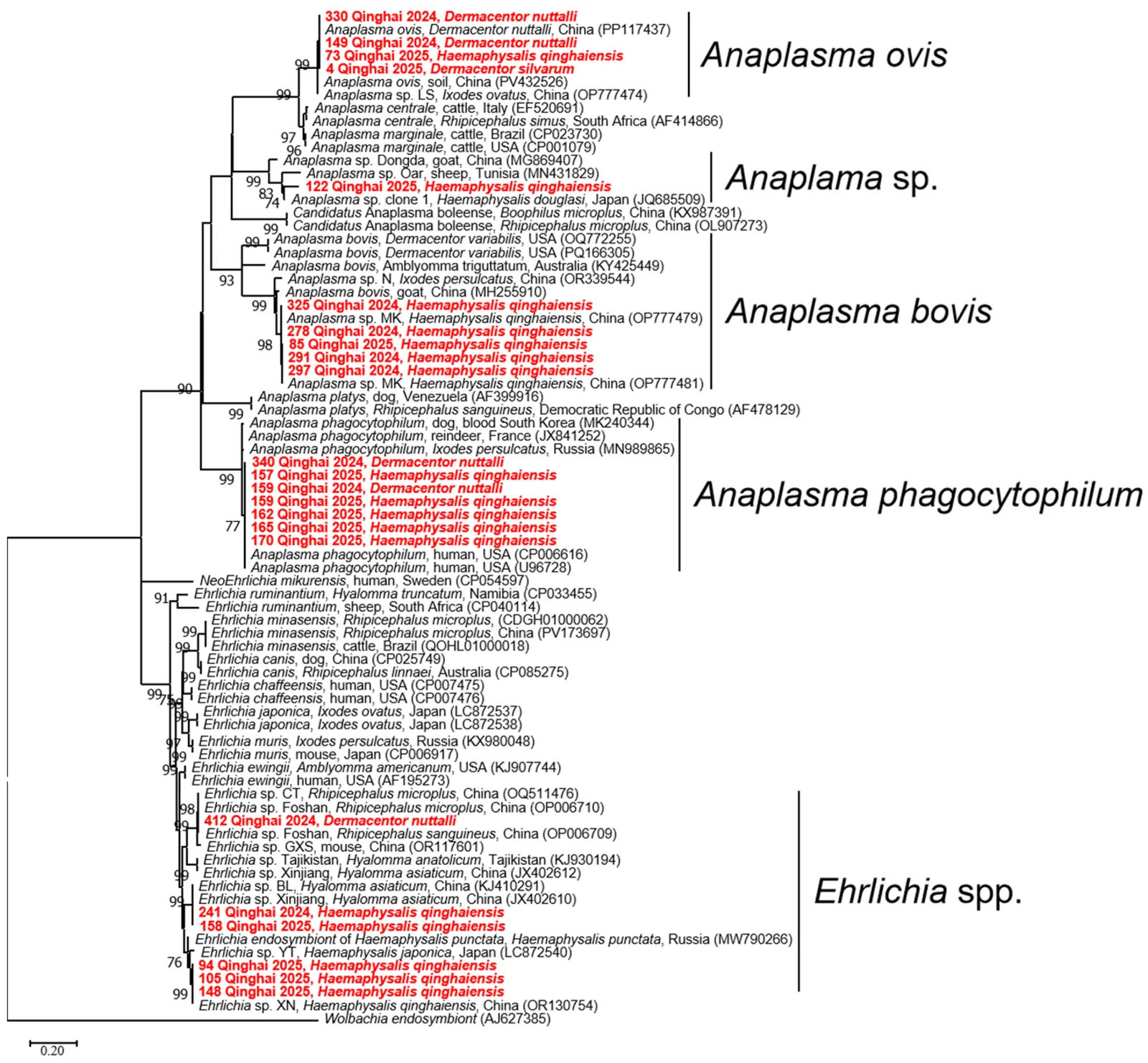
Phylogenetic analysis of Anaplasmataceae *groEL* gene sequences. A maximum likelihood tree was constructed using *groEL* sequences obtained in the present study, together with reference sequences from GenBank. Sequences generated in the present study, shown in red, clustered with *Anaplasma ovis*, *Anaplasma bovis*, *Anaplasma phagocytophilum*, uncultured *Anaplasma* sp., and *Ehrlichia* spp.

**Figure 4:**
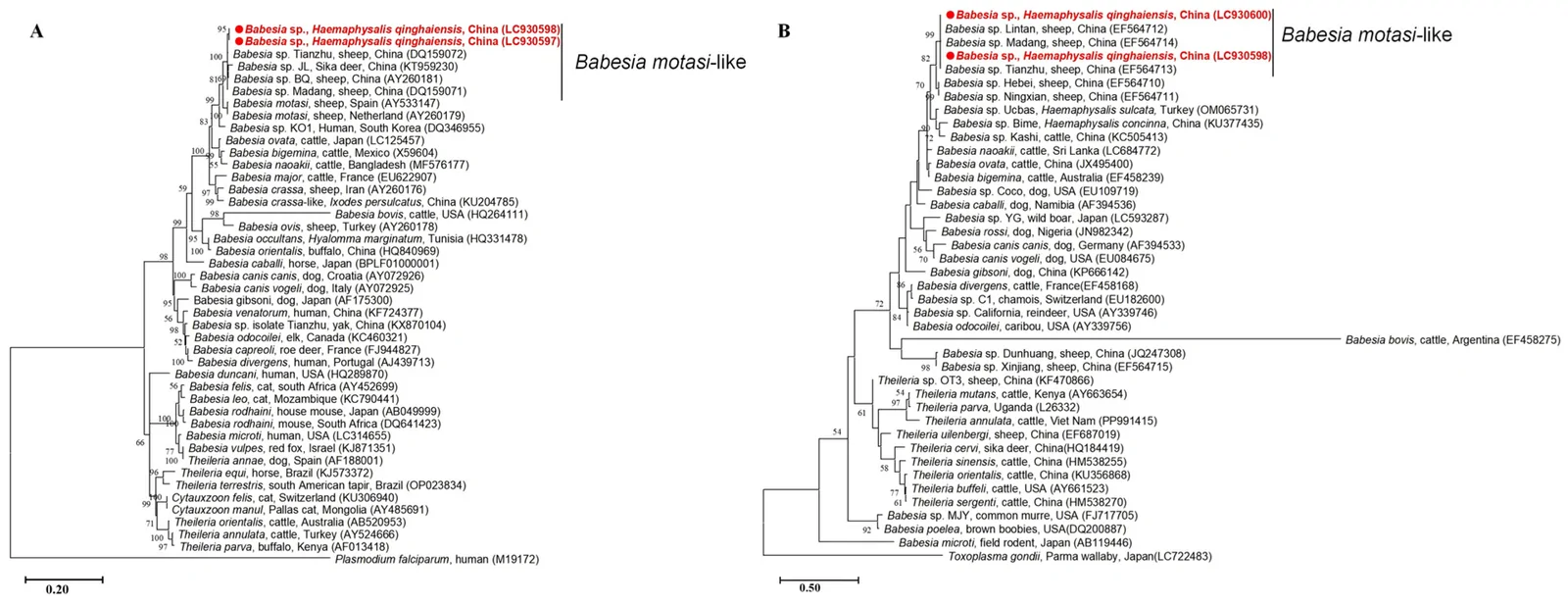
Phylogenetic analysis of *Babesia* spp. Two maximum likelihood trees were constructed using 18S rRNA (panel (**A**)) and Internal Transcribed Spacer 2 (ITS2) (panel (**B**)) sequences generated in the present study, together with reference sequences from GenBank. Sequences obtained in the present study, shown in red, clustered within the *Babesia motasi*-like group.

Sequence and phylogenetic analyses revealed differences in pathogen-related sequence diversity across altitude zones and tick species (Figure 2, Figure 3 and Figure 4). Among the *Rickettsia*-positive samples, *gltA* sequences shared high identity with those from five taxa: *R. raoultii* (98.70–100.00% identity with GenBank accession no. MF511244), *R. slovaca* (100.00% with AY129301), *Rickettsia* sp. HHMJ7 (100.00% with KC566999), *Rickettsia* sp. Osumi (100.00% with LC786368), and *Rickettsia* sp. DT-UR (99.74% with MN630883). Phylogenetic analysis further supported these findings, with the obtained sequences clustering within the corresponding *Rickettsia* clades (Figure 2). Newly generated *gltA* sequences corresponding to the above taxa were deposited in GenBank under accession nos. LC930457-LC930573. The high-altitude zone showed greater *Rickettsia* diversity, with sequences related to all five taxa detected, whereas only three taxa (*R. raoultii*, *R. slovaca*, and *Rickettsia* sp. HHMJ7) were detected in the very high-altitude zone.

At the tick species level, *H. qinghaiensis* showed the highest *Rickettsia* diversity (Table 3), harboring sequences related to all five taxa, including *R. raoultii* (*n* = 1), *R. slovaca* (*n* = 11), *Rickettsia* sp. HHMJ7 (*n* = 19), *Rickettsia* sp. Osumi (*n* = 27), and *Rickettsia* sp. DT-UR (*n* = 2). *Dermacentor nuttalli* yielded sequences related to four taxa, including *R. raoultii* (*n* = 16), *R. slovaca* (*n* = 2), and *Rickettsia* sp. HHMJ7 (*n* = 33), and *Rickettsia* sp. Osumi (*n* = 2). *Dermacentor* silvarum yielded sequences related to *R. raoultii* (*n* = 1) and *Rickettsia* sp. HHMJ7 (*n* = 2), whereas *D. niveus* yielded only a sequence related to *R. raoultii* (*n* = 1).

### 3.4. Anaplasmataceae Species Detected in Collected Ticks

Among Anaplasmataceae-positive samples, *groEL* sequences showed high identity to those from *A. phagocytophilum* (100.00% with U96728), *A. bovis* (99.45–99.72% with OK625727), *A. ovis* (99.73–100.00% with PP117437), one uncultured *Anaplasma* sp. (94.20% with JQ685509), *Ehrlichia* sp. XN (100.00% with OR130754), *Ehrlichia* sp. BL116 (99.73–100.00% with KJ410291), and *Ehrlichia* sp. isolate Foshan8 (100.00% with OP006710). These sequences clustered within their respective Anaplasmataceae clades in the phylogenetic tree (Figure 3). Newly generated *groEL* sequences corresponding to the above taxa were deposited in GenBank under accession nos. LC930574-LC930596. Similar to *Rickettsia*, greater Anaplasmataceae diversity was observed in the high-altitude zone, where sequences related to six taxa were detected, whereas only three taxa (*A. phagocytophilum*, *A. ovis*, and *Ehrlichia* sp. isolate Foshan8) were detected in the very high-altitude zone.

At the tick species level, *H. qinghaiensis* showed the highest Anaplasmataceae diversity (Table 3), yielding sequences related to *A. phagocytophilum* (*n* = 5), *A. bovis* (*n* = 5), *A. ovis* (*n* = 1), one uncultured *Anaplasma* sp. (*n* = 1), *Ehrlichia* sp. XN (*n* = 3), and *Ehrlichia* sp. BL116 (*n* = 2). *Dermacentor nuttalli* yielded sequences related to *A. phagocytophilum* (*n* = 2), *A. ovis* (*n* = 2), and *Ehrlichia* sp. isolate Foshan8 (*n* = 1), whereas *D. silvarum* yielded one sequence related to *A. ovis*.

### 3.5. Babesia Species Detected in Collected Ticks

*Babesia* 18S rRNA sequences were detected only in the high-altitude zone, and exclusively in two *H. qinghaiensis*. Both sequences showed 99.67% identity with *Babesia* sp. Madang-2005 (GenBank accession no. DQ159071), and clustered within the *B. motasi*-like group (Figure 4). Analysis of ITS2 further supported this classification, with the obtained sequence showing 99.69% identity with *Babesia* sp. Madang (EF564714). Newly generated *Babesia* 18S rRNA and ITS2 sequences were deposited in GenBank under accession nos. LC930597-LC930598 and LC930599-LC930600, respectively.

### 3.6. Co-Infections

Co-infections involving *Rickettsia* and Anaplasmataceae were detected only in five ticks, including four *H. qinghaiensis* (two females and two nymphs) and one female *D. silvarum*, from the high-altitude zone. Specifically, four *H. qinghaiensis* ticks infected with *Rickettsia* sp. were co-infected with *Ehrlichia* sp., *A. bovis*, *Anaplasma* sp., or *A. phagocytophilum*. In addition, the *D. silvarum* tick was co-infected with *Rickettsia* sp. and *A. ovis*.

## 4. Discussion

The present study investigated questing tick species and associated zoonotic pathogens in the high- and very high-altitude zones of QTPA, providing insights into the composition of tick species and pathogens that they harbor in this climatically unique ecosystem in China. Two principal and novel findings emerged from this study. First, tick species composition differed markedly between the two altitude zones, with *H. qinghaiensis* predominating in the high-altitude zone and *D. nuttalli* predominating in the very high-altitude zone. Second, pathogen-group diversity was greater in the high-altitude zone, where *Rickettsia*, Anaplasmataceae, and *Babesia* DNA were detected, whereas only *Rickettsia* and Anaplasmataceae DNA were detected in the very high-altitude zone.

Clear altitude-associated differences were observed in tick species composition. *Haemaphysalis qinghaiensis* dominated the high-altitude zone, whereas *Dermacentor nuttalli* was the predominant species in the very high-altitude zone. This pattern likely reflects ecological adaptation to contrasting environmental conditions. *Dermacentor nuttalli* is widely distributed across eastern Siberia, Mongolia, and northern China and is associated with steppe and grassland environments [38,39]. It parasitizes a wide range of domestic and wild mammals and can also feed on humans, indicating broad host associations and epidemiological importance [38,39]. Its predominance in very high-altitude zones in the present study may be associated with its tolerance of the relatively cold and dry conditions of high-elevation plateau environments [38]. In contrast, *H. qinghaiensis* is an endemic tick species distributed mainly in the western plateau regions of China, including Qinghai, Gansu, Sichuan, and Tibet [40]. It is closely associated with livestock, particularly yaks, sheep, goats, and cattle, which serve as important hosts [40]. Its close association with domestic ruminants may contribute to its high abundance in the high-altitude zone surveyed in the present study, where livestock grazing is widespread. Thus, differences in host associations and ecological distributions may partly explain the contrasting distributions of *H. qinghaiensis* and *D. nuttalli* along the altitude gradient. The sporadic occurrence of *D. silvarum* and *D. niveus* in both zones may reflect incidental introduction through livestock movement or limited adaptation to the surveyed ecological settings. Overall, our findings indicate that environmental filtering associated with altitude plays a key role in shaping the composition of the tick community in the QTPA.

DNAs of several pathogens were detected in multiple tick species collected in both altitude zones, indicating active circulation of tick-borne microorganisms in the surveyed areas. Sequences closely related to zoonotic or potentially zoonotic agents, including *R. raoultii*, *R. slovaca*, *A. phagocytophilum*, and *A. bovis*, were identified. These pathogens have previously been reported in ticks and animal hosts in China and are recognized as pathogens of veterinary and public health importance [10,11,41,42]. Their detection in questing ticks supports the potential role of *H. qinghaiensis*, *D. nuttalli*, and *D. silvarum* as candidate vectors in the QTPA, consistent with previous reports from other regions [43]. However, although *A. phagocytophilum* DNA was detected in *H. qinghaiensis* and *D. nuttalli*, *Ixodes* species are currently regarded as the principal competent vectors of *A. phagocytophilum*. Therefore, the detection of *A. phagocytophilum* sequences in *H. qinghaiensis* and *D. nuttalli* in the present study should be interpreted as detection of pathogen-associated DNA rather than definitive evidence of vector competence. In addition, purified DNA from the *A. phagocytophilum* HZ strain was used as the positive control for the *groEL* PCR assay. Although appropriate negative controls were included and no evidence of contamination was observed, inadvertent contamination from the positive control cannot be completely excluded.

*Babesia* sequences related to the *B. motasi*-like group were detected in two *H. qinghaiensis* samples. Although *B. motasi*-like spp. have occasionally been reported in humans [44], the zoonotic significance of the organism detected in the present study remains unclear. In the present study, pathogen profiles differed between the two predominant tick species, with *H. qinghaiensis* harboring the sequences related to *Rickettsia*, Anaplasmataceae, and *Babesia*, whereas *D. nuttalli* was associated only with *Rickettsia*- and Anaplasmataceae-related sequences. However, the relatively low positivity rates and limited sample size for several detected taxa preclude further interpretation regarding species-specific vector-pathogen associations.

The present study has several limitations. First, only high- and very high-altitude zones were surveyed, whereas extreme altitude and death zone areas were not included because of accessibility and logistical constraints. Therefore, the full altitudinal distribution of ticks and associated pathogens remains unclear across the QTPA. Future studies involving broader geographic and altitudinal sampling, together with investigation of host animals and human infections, will be important for better understanding tick-borne pathogen circulation in the QTPA. Second, host animals were not sampled concurrently, limiting assessment of the host species involved in pathogen maintenance and transmission. In addition, only nymphal and adult ticks were collected, limiting assessment of pathogen acquisition and persistence across the complete tick life cycle. Third, relatively short PCR fragments and single-locus PCR and sequencing were used for pathogen identification, without confirmation using additional molecular markers. In particular, the *gltA* and *groEL* fragments may provide limited resolution among closely related *Rickettsia* and Anaplasmataceae taxa. Moreover, several obtained sequences clustered with previously reported uncultured *Rickettsia*, *Anaplasma*, and *Ehrlichia* species in the phylogenetic trees. The pathogenicity and zoonotic potential of these uncultured taxa also remain unclear, warranting further investigation to better understand their epidemiological significance in the QTPA of China. Finally, PCR detection alone cannot demonstrate vector competence, because the presence of pathogen DNA does not establish pathogen viability, replication, persistence, or transmission by the tick. Further experimental studies will therefore be required to determine whether the tick species examined in this study are competent vectors.

The public health implications of tick-borne pathogens in the QTPA may differ, depending not only on the prevalence of pathogens, but also on the dominant tick species in each ecological zone. In the present study, the high-altitude zone was dominated by *H. qinghaiensis*, which primarily infests livestock in China. In contrast, the very high-altitude zone was dominated by *D. nuttalli*, which has been frequently reported parasitizing both livestock and humans, and implicated in transmission of several zoonotic pathogens, particularly spotted fever group *Rickettsia* [39]. Therefore, the predominance of *D. nuttalli* may represent an important public health concern in the very high-altitude zone. Since limited medical accessibility and diagnostic capacity may contribute to underrecognition or underreporting of tick-borne infections in remote plateau areas [45], enhanced surveillance of human infections is a priority in the QTPA of China.

## 5. Conclusions

This study demonstrated that tick species composition and associated zoonotic pathogens differ between the high- and very high-altitude zones of the QTPA. *Haemaphysalis qinghaiensis* predominated in the high-altitude zone, whereas *D. nuttalli* predominated in the very high-altitude zone. Although ticks from both zones tested positive for several zoonotic *Rickettsia* and *Anaplasma* species, pathogen diversity was greater in the high-altitude zone. These findings indicate an association between altitude zone and tick community composition, which may in turn have contributed to differences in the distribution of zoonotic tick-borne pathogens across altitude zones. They also highlight the potential risk of human exposure in the high- and very high-altitude zones of the QTPA, particularly in the very high-altitude zone where the human-biting tick *D. nuttalli* is predominant.

These findings provide a baseline for understanding the ecological distribution of questing ticks and associated tick-borne pathogens across different altitude zones of the QTPA. The marked differences in tick community composition and pathogen profiles highlight the importance of considering altitude and tick ecology in future surveillance of tick-borne pathogens in this region. However, pathogen DNA detection does not establish vector competence or zoonotic transmission. Further studies integrating tick, livestock, wildlife, and human samples, together with multilocus or genomic characterization and vector competence investigations, are warranted to clarify pathogen transmission cycles and their veterinary and public health significance in the QTPA.

## Figures and Tables

**Table 1:** PCR Primers used in the present study.

Pathogens	Target Sequences	Primer Sequence (5′-3′)		Amplicon Size (bp)	References
*Rickettsia*	*gltA*	F ^a^	ATGACCAATGAAAATAATAAT	~1290	[26]
		R ^b^	CTTATACTCTCTATGTACA		
		F	GGGGGCCTGCTCACGGCGG	~381	
		R	ATTGCAAAAAGTACAGTGAACA		
Anaplasmataceae	*groEL*	F	GAAGATGCWGTWGGWTGTACKGC	~670	[27]
		R	AGMGCTTCWCCTTCWACRTCYTC		
		F	ATTACTCAGAGTGCTTCTCARTG	~444	
		R	TGCATACCRTCAGTYTTTTCAAC		
*B. microti* and *B. duncani*	18S rRNA	F	TGTAATTGGAATGATGGGAATC	555–564	[30]
		R	CAAGGTGCTGAAGGAGTCGTTT		
*B. divergens*, *B. venatorum*, and *B. odocoilei*	18S rRNA	F	AATCGCAATTTTTTGCGATGGA	910–911	
		R	ATCCTAACATTGTCTGGACCTG		
*B. crassa*-like species and *Babesia* sp. KO1	18S rRNA	F	TGCGAATCGCGTTAGCGATGTT	1208–1215	
		R	ATCAATCCCCGACACGATGCAC		
*Babesia* spp.	ITS2	F	GGATGTCTTGGCTCACACAAC	~361	Newly designed
		R	TAAATTCAGCGGATAGCCTCACC		

*B. microti*, *Babesia microti*; *B. duncani*, *Babesia duncani*; *B. divergens*, *Babesia divergens*; *B. venatorum*, *Babesia venatorum*; *B. odocoilei*, *Babesia odocoilei*. ^a^ F, forward primer. ^b^ R, reverse primer.

**Table 2:** PCR detection of *Rickettsia*, Anaplasmataceae, and *Babesia* in questing ticks at different altitudes in QTPA.

Plateau Zones	Tick Species		Tick No. (% ^a^)	No. Positive (%)		
				*Rickettsia*	Anaplasmataceae	*Babesia*
High altitude	*H. qinghaiensis*	Male	113 (31.0)	20 (17.7)	5 (4.4)	1 (0.9)
		Female	143 (39.3)	23 (16.1)	5 (3.5)	1 (0.7)
		Nymph	60 (16.5)	17 (28.3)	7 (11.7)	0
		**Total**	**316 (** **86.8** **)**	**60 (19.0)**	**17 (5.4)**	**2 (0.6)**
	*D. nuttalli*	Male	14 (3.8)	4 (28.6)	0	0
		Female	23 (6.3)	3 (13.0)	0	0
		**Total**	**37 (10.2)**	**7 (18.9)**	**0**	**0**
	*D. silvarum*	Male	4 (1.1)	1 (25.0)	0	0
		Female	5 (1.4)	2 (40.0)	1 (20.0)	0
		**Total**	**9 (2.5)**	**3 (33.3)**	**1 (11.1)**	**0**
	*D. niveus*	Male	2 (0.5)	1 (50.0)	0	0
		Female	0	0	0	0
		**Total**	**2 (0.5)**	**1 (50.0)**	**0**	**0**
	**Total**		**364**	**71 (19.5)**	**18 (4.9)**	**2 (0.5)**
Very high altitude	*D. nuttalli*	Male	217 (32.8)	18 (8.3)	2 (0.9)	0
		Female	431 (65.2)	28 (6.5)	3 (0.7)	0
		**Total**	**648 (98.0)**	**46 (7.1)**	**5 (0.8)**	**0**
	*D. silvarum*	Male	4 (0.6)	0	0	0
		Female	7 (1.1)	0	0	0
		**Total**	**11 (1.7)**	**0**	**0**	**0**
	*D. niveus*	Male	0	0	0	0
		Female	2 (0.3)	0	0	0
		**Total**	**2 (0.3)**	**0**	**0**	**0**
	**Total**		**661**	**46 (7.0)**	**5 (0.8)**	**0**
**Total**			**1025**	**117 (11.41)**	**23 (2.24)**	**2 (0.20)**

*H. qinghaiensis*, *Haemaphysalis qinghaiensis*; *D. nuttalli*, *Dermacentor nuttalli*; *D. silvarum*, *Dermacentor silvarum*; *D. niveus*, *Dermacentor niveus*. ^a^ The percentage of each tick species, calculated based on the total number of ticks collected in high- and very-high altitude zones.

**Table 3:** Summary of tick-borne pathogens in questing ticks from the QTPA.

Pathogens		No. Positive (%)									
		High Altitude					Very High Altitude				
		*H. qinghaiensis* (*n* = 316)	*D. nuttalli* (*n* = 37)	*D. silvarum* (*n* = 9)	*D. niveus* (*n* = 2)	Total (*n* = 364)	*D. nuttalli* (*n* = 648)	*D. silvarum* (*n* = 11)	*D. niveus* (*n* = 2)	Total (*n* = 661)	Total (*n* = 1025)
***Rickettsia***	*R. raoultii*	1 (0.3)	1 (2.7)	1 (11.1)	1 (50.0)	4 (1.1)	15 (2.3)	0	0	15 (2.3)	19 (1.9)
	*R. slovaca*	11 (3.5)	0	0	0	11 (3.0)	2 (0.3)	0	0	2 (0.3)	13 (1.3)
	*Rickettsia* spp.	48 (15.2)	6 (16.2)	2 (22.2)	0	56 (15.4)	29 (4.5)	0	0	29 (4.4)	85 (8.3)
**Anaplasmataceae**	*A. phagocytophilum*	5 (1.6)	0	0	0	5 (1.4)	2 (0.3)	0	0	2 (0.3)	7 (0.7)
	*A. bovis*	5 (1.6)	0	0	0	5 (1.4)	0	0	0	0	5 (0.5)
	*A. ovis*	1 (0.3)	0	1 (11.1)	0	2 (0.5)	2 (0.3)	0	0	2 (0.3)	4 (0.4)
	*Anaplasma* sp.	1 (0.3)	0	0	0	1 (0.3)	0	0	0	0	1 (0.1)
	*Ehrlichia* spp.	5 (1.6)	0	0	0	5 (1.4)	1 (0.2)	0	0	1 (0.2)	6 (0.6)
***Babesia***	*B. motasi*-like	2 (0.6)	0	0	0	2 (0.5)	0	0	0	0	2 (0.2)

*H. qinghaiensis*, *Haemaphysalis qinghaiensis*; *D. nuttalli*, *Dermacentor nuttalli*; *D. silvarum*, *Dermacentor silvarum*; *D. niveus*, *Dermacentor niveus*; *R. raoultii*, *Rickettsia raoultii*; *R. slovaca*, *Rickettsia slovaca*; *A. phagocytophilum*, *Anaplasma phagocytophilum*; *A. bovis*, *Anaplasma bovis*; *A. ovis*, *Anaplasma ovis*; *B. motasi*-like, *Babesia motasi*-like.

## Data Availability

The original contributions presented in this study are included in the article/Supplementary Material. Further inquiries can be directed to the corresponding authors.

## Supplementary Materials

The following supporting information can be downloaded at: https://www.mdpi.com/article/10.3390/ani16182927/s1, Figure S1: Species composition, developmental stages, and relative abundance of questing ticks collected from the northeastern Qinghai-Tibetan Plateau; Table S1: Accession numbers of DNA sequences from this study deposited in GenBank.

## Abbreviations

The following abbreviations are used in this manuscript:

*A. bovis*	*Anaplasma bovis*
*A. ovis*	*Anaplasma ovis*
*A. phagocytophilum*	*Anaplasma phagocytophilum*
*B. crassa*-like	*Babesia crassa*-like
*B. divergens*	*Babesia divergens*
*B. duncani*	*Babesia duncani*
*B. microti*	*Babesia microti*
*B. motasi*-like	*Babesia motasi*-like
*B. odocoilei*	*Babesia odocoilei*
*B. venatorum*	*Babesia venatorum*
*D. niveus*	*Dermacentor niveus*
*D. nuttalli*	*Dermacentor nuttalli*
*D. silvarum*	*Dermacentor silvarum*
*E. chaffeensis*	*Ehrlichia chaffeensis*
*E. ewingii*	*Ehrlichia ewingii*
*H. qinghaiensis*	*Haemaphysalis qinghaiensis*
QTPA	Qinghai-Tibetan Plateau area
*R. raoultii*	*Rickettsia raoultii*
*R. slovaca*	*Rickettsia slovaca*
