# Supplementary material for: Epidemiological Survey of Tick-Borne Zoonotic Pathogens in Questing Ticks from the Qinghai-Tibetan Plateau of China
Source: Animals (Basel). 2026 Sep 17;16(18):2927. doi: 10.3390/ani16182927 (PMC13603127; doi:10.3390/ani16182927)

Table S1. Accession numbers of DNA sequences from this study deposited in GenBank.

Pathogens	Accession number	Sequence length analyzed	Query Cover	E value	Perident	Reference Accession	Host
Rickettsia raoultii	LC930457-LC930475	382	100%	0	98.70-100.00	MF511244	*Ixodes persulcatus*
Rickettsia slovaca	LC930476-LC930488	382	100%	0	100	AY129301	*Dermacentor marginatus*
Rickettsia spps.	LC930489-LC930542	382	100%	0	100	KC566999	*Dermacentor silvarum*
	LC930543-LC930571	382	100%	0	100	LC786368	Haemaphysalis sp.
	LC930572-LC930573	382	100%	0	99.74	MN630883	*Haemaphysalis longicornis*
Anaplasma phagocytophilum	LC930574-LC930580	362	100%	0	100	U96728	human
Anaplasma bovis	LC930581-LC930585	362	100%	0	99.45-99.72	OK625727	yak
Anaplasma *ovis*	LC930586-LC930589	365	100%	0	99.73-100.00	PP117437	*Dermacentor nuttalli*
Anaplasma sp.	LC930590	362	100%	9.00E-153	94.2	JQ685509	*Haemaphysalis douglasi*
Ehrlichia spps.	LC930591-LC930593	365	100%	0	100	OR130754	*Haemaphysalis qinghaiensis*
	LC930594-LC930595	365	100%	0	99.73-100.00	KJ410291	*Hyalomma asiaticum*

	LC930596	365	100%	0	100	OP006710	*Rhipicephalus microplus*
Babesia sp. (18S)	LC930597-LC930598	1,229	100%	0	99.67	DQ159071	sheep
Babesia sp. (ITS2)	LC930599-LC930600	367	89%	1.00E-165	100	EF564713	sheep

Figure S1. Species composition, developmental stages, and relative abundance of questing ticks collected from the northeastern Qinghai-Tibetan Plateau.

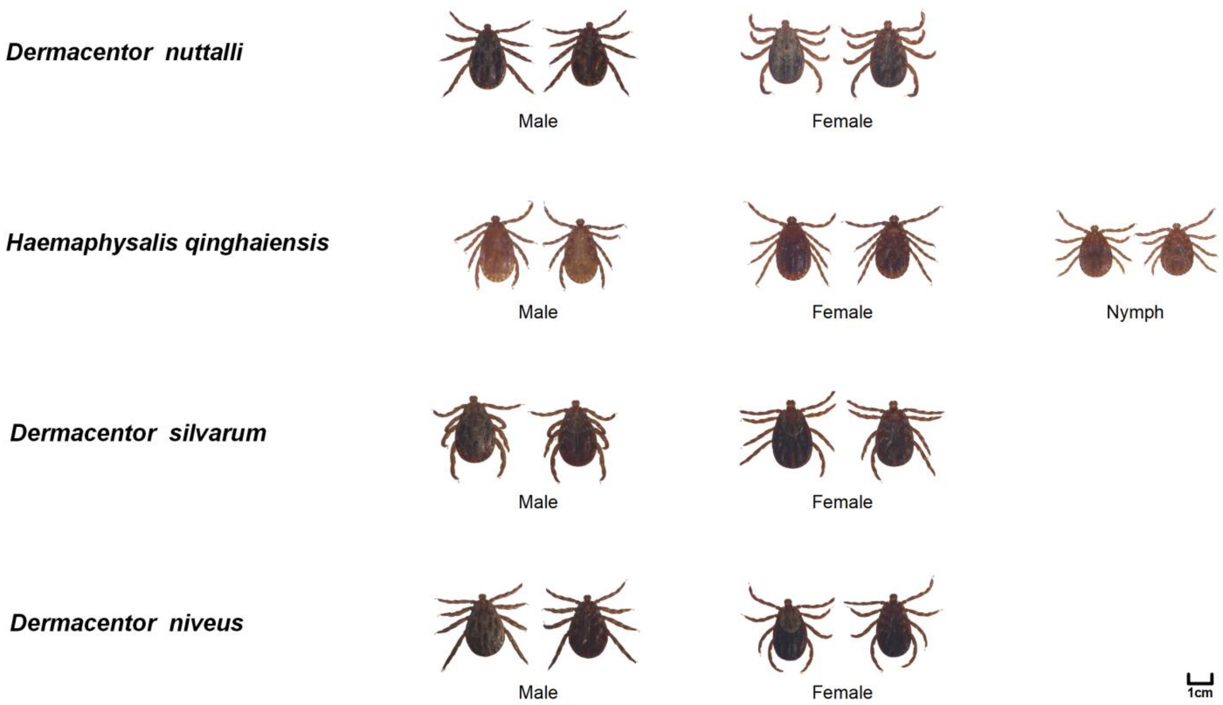

Supplement: Supplementary file 1 [file animals-16-02927-s001.zip › animals-4496302-supplementary.pdf]
